# A nonrandomized phase I and biomarker trial of regorafenib in advanced myeloid malignancies

**DOI:** 10.1002/jha2.408

**Published:** 2022-03-06

**Authors:** Joan How, Siyang Ren, Jennifer Lombardi‐Story, Meghan Bergeron, Julia Foster, Phillip C. Amrein, Andrew M. Brunner, Amir T. Fathi, Hanno Hock, Anna Khachatryan, Hiroto Kikuchi, Mei Rosa Ng, Jenna Moran, Rupa Narayan, Donna Neuberg, Aura Ramos, Tina Som, Meghan Vartanian, Yi‐Bin Chen, Dan G. Duda, Gabriela S. Hobbs

**Affiliations:** ^1^ Division of Hematology Brigham and Women's Hospital Harvard Medical School Boston Massachusetts USA; ^2^ Department of Data Sciences Dana‐Farber Cancer Institute Boston Massachusetts USA; ^3^ Department of Medical Oncology Massachusetts General Hospital Harvard Medical School Boston Massachusetts USA; ^4^ Department of Radiation Oncology Massachusetts General Hospital Harvard Medical School Boston Massachusetts USA

**Keywords:** antiangiogenesis, clinical trials, myeloid leukaemia

## Abstract

We conducted a single‐center, open‐label, dose escalation, and expansion phase I trial of the antiangiogenic multikinase inhibitor regorafenib in patients with advanced myeloid neoplasms. We enrolled 16 patients with relapsed/refractory acute myeloid leukemia (AML), myeloproliferative neoplasms (MPN), chronic myelomonocytic leukemia (CMML), or myelodysplastic syndrome (MDS). A 3 + 3 dose escalation design was used with two planned dose levels (120 or 160 mg daily) and one de‐escalation level (80 mg daily). An additional 10 patients were treated on an expansion cohort. The recommended phase two dose of regorafenib was 160 mg daily, with no dose‐limiting toxicities. The best overall disease response by International Working Group criteria included one partial and stable disease in 11 patients. Tissue studies indicated no change in Ras/mitogen‐activated protein kinase (MAPK) pathway activation in responders. Pharmacodynamic changes in plasma VEGF, PlGF, and sVEGFR2 were detected during treatment. Baseline proinflammatory and angiogenic cytokine levels were not associated with clinical response. Single‐agent regorafenib demonstrated an acceptable safety profile in relapsed/refractory myeloid malignancy patients. Most patients achieved stable disease, with modest improvements in cell counts in some MDS patients. Biomarker studies were consistent with on‐target effects of regorafenib on angiogenesis. Future studies should investigate the role of regorafenib in combination therapy approaches.

## 1 INTRODUCTION

Vascular endothelial growth factor (VEGF) pathway inhibitors are standard therapies for many solid cancers. The bone marrow microenvironment is an important regulator of hematopoiesis in hematologic malignancies. Malignant hematopoietic cells consume high levels of oxygen and secrete pro‐angiogenic molecules such as VEGF to support leukemic growth [1]. Microvessel density increases in the bone marrow of patients with myeloid malignancies including acute myeloid leukemia (AML) [2, 3, 4], chronic myelomonocytic leukemia (CMML) [2], myelodysplastic syndrome (MDS) [2, 5], and myelofibrosis (MF) [6]. Within AML specifically, increased bone marrow angiogenesis is associated with decreased overall survival [7], and microvessel density reverts to levels similar to controls following induction chemotherapy [8]. Angiogenesis and VEGF expression are increased in patients with myeloproliferative neoplasms (MPNs), especially in MF [9], and in advanced MDS and CMML [10].

VEGF inhibitors have been previously evaluated in myeloid malignancies given the essential role of this pathway in tumor angiogenesis. A phase 2 trial of bevacizumab, a recombinant monoclonal antibody targeting VEGF, with standard induction chemotherapy in newly diagnosed elderly AML patients showed no improvements in clinical outcomes [11]. However, a separate phase 2 trial in relapsed/refractory AML utilizing a time‐sequential strategy of chemotherapy followed by bevacizumab yielded a modest complete remission (CR) rate of 33% [12]. A phase 2 study of MF patients receiving bevacizumab monotherapy demonstrated significant toxicity such that the trial was closed early, with no objective responses observed [13]. Aflibercept, a decoy receptor and VEGF trap, was tested in a phase 2 trial of MDS and MDS/MPN overlap patients who have failed hypomethylating agents, and was also ultimately halted early due to lack of efficacy [14].

These disappointing results may be related to the limitations of more selective angiogenesis inhibitors. A variety of mutations have been identified in the pathogenesis of myeloid malignancies, which result in activation of transcription or growth factor receptors such as but not limited to, RAS/RAF/Mitogen‐activated protein kinase/extra‐cellular signal regulated kinase (ERK) kinase (MEK)/ERK, Janus kinase (JAK)‐signal transducer and activator of transcription, and Fms‐like tyrosine kinase 3 (FLT3) pathways [15, 16, 17]. Molecularly targeted therapies, including FLT3 and isocitrate dehydrogenase inhibition, have been developed and represent a major step forward in treatment [18, 19]. However, these approaches usually have limited responses as single agents, in part due to the clonal heterogeneity of diseases and the existence of redundant pathways leading to oncogenesis. Regorafenib is a multikinase inhibitor that targets angiogenic, stromal, and oncogenic kinases and is approved for the treatment of advanced hepatocellular and colorectal carcinomas. As a result of regorafenib's broad inhibition of kinases and its effects on angiogenesis, regorafenib has the potential to overcome the limitations of more selective/specific VEGF inhibitors in advanced myeloid malignancies.

## 2 PATIENTS AND METHODS

### 2.1 Study design and procedures

We conducted a single‐center, open‐label, dose escalation, and expansion phase I trial in patients with relapsed/refractory AML, MPN, CMML, or MDS to assess the safety, tolerability, and preliminary efficacy of regorafenib. A 3+3 dose escalation design was used with two planned dose levels (120 mg or 160 mg daily), as well as one de‐escalation level (80 mg daily) if needed. Three to six patients were evaluated per dose level, and dose escalation continued until the maximum tolerated dose (MTD) was identified, defined as the highest dose level where 0/3 or 1/6 patients experienced a dose‐limiting toxicity (DLT). For the purposes of dose escalation, a DLT was defined as grade ≥3 toxicity occurring within the first 28 days after initiation of treatment and unrelated to the underlying myeloid neoplasm. Toxicity was graded using Common Terminology Criteria for Adverse Events version 4.0. In the expansion phase of the trial, an additional 10 patients were enrolled at the MTD. The primary objective of this study was to assess the safety and tolerability of regorafenib in this patient population and to establish the recommended phase 2 dose (RP2D). The secondary objective was to assess the pharmacodynamics of regorafenib via changes in known regorafenib targets and to assess treatment response to regorafenib.

Treatment consisted of regorafenib at the starting dose level of 120 mg daily for 3 weeks on and 1 week off and administered on repeating cycles of 28 days. Patients were followed and treated on an outpatient basis for physical examination, standard laboratory testing, and adverse event evaluation. All patients received a baseline bone marrow aspiration and biopsy within 28 days of enrollment and repeated at 1, 3, 6, and 12 months and if there was concern for disease progression or relapse. Correlative testing occurred at time points as described below. Patients were continued on regorafenib if they derived clinical benefit and were tolerating therapy. All patients who received a dose of regorafenib were included in analysis.

The protocol was approved by the institutional review board and compliant with the Declaration of Helsinki and guidelines on Good Clinical Practice. All patients provided informed consent.

### 2.2 Patient eligibility

Eligible patients were adults (≥18 years) with a diagnosis of advanced myeloid malignancy, as defined by: relapsed/refractory AML with failure of at least one line of prior therapy; patients with MDS and CMML that have failed hypomethylating agents; or patients with MF or unclassified MPN on maximal tolerated ruxolitinib with persistent residual symptoms, splenomegaly, or inadequately controlled blood counts. Patients were required to have adequate organ function and Eastern Cooperative Oncology Group (ECOG) performance status of 0–2.

Exclusion criteria included prior antineoplastic therapy in the last 14 days (except hydroxyurea); uncontrolled hypertension (systolic pressure >140 mmHg or diastolic pressure >90 mmHg on repeated measurement despite optimal medical management); history of arterial or venous thromboembolism within the last 6 months of informed consent; coagulopathy or other bleeding diathesis; seizure disorder; active and significant heart disease, presence of nonhealing wound, ulcer, or fracture; interstitial lung disease; and pregnancy.

### 2.3 Correlative studies

Exploratory analyses of biomarkers were evaluated in participants at baseline (C1D1 prior to first treatment dose) and on C1D8, C1D15, and C2D1. Whole blood was centrifuged and separated into plasma and cellular components. The following proangiogenic and proinflammatory biomarkers were performed on plasma with the Human Angiogenesis Panel 1 V‐PLEX kit (Meso‐Scale Discovery, Gaithersburg, MD, #K15190D) for VEGF, bFGF, sFLT‐1, PIGF, sTie‐2, VEGF‐C, and VEGF‐D; the Human Pro‐Inflammatory Panel 1 V‐PLEX (Meso‐Scale Discovery, #K15049D) for IFN‐γ, IL‐10, IL‐12p70, IL‐13, IL‐1β, IL‐2, IL‐4, IL‐6, interleukin 8 (IL‐8), and TNF‐α; the V‐plex Human IP‐10 kit (Meso‐Scale Discovery, #K151NVD) for IP‐10 and VEGFR2 (R&D Systems, Minneapolis, MN, #DVR200). All samples were run in duplicate in the CLIA certified core of the Steele Laboratories at Massachusetts General Hospital (MGH). The fraction of lymphocyte and myeloid subtypes from the total circulating mononuclear cells were counted by flow cytometry using the following markers: CD3, CD4, CD8, CD25, CD45, CD56, and CD127 (BD Biosciences). Protein extract was obtained from bone marrow aspirates and Peripheral blood mononuclear cells (PBMCs) from selected patients with mutations in MAPK pathway and analyzed by Western blotting for total and phosphorylated ERK.

### 2.4 Statistical analysis

The primary objective of this study was to evaluate the safety and tolerability of regorafenib in patients with advanced myeloid malignancies. All patients who received at least one dose of regorafenib were included for analysis. DLTs were assessed during cycle 1, and rates of toxicity were summarized descriptively.

The secondary objectives of this study were to assess responses to regorafenib and evaluate potential pharmacodynamic biomarkers of regorafenib by measuring baseline and changes after treatment in known regorafenib targets. Disease response was defined by International Working Group (IWG) criteria for AML, MDS, and CMML patients respectively and IWG MPNs Research and Treatment (MRT) criteria for MF [20, 21, 22]. Bone marrow biopsies for disease response assessment were performed at 1, 3, 6, 9, and 12 months and summarized with descriptive statistics.

For biomarker analysis, variables were summarized as median and interquartile ranges at each timepoint. Statistical analysis was performed using R 4.0. A Wilcoxon signed‐rank test was used to test the paired differences in biomarkers from C1D1 to C1D8. Plasma biomarkers were excluded if more than 50% of values were out of the range of detection. For the purposes of plasma biomarker testing, responders were defined as patients who obtained a partial response or had stable disease for at least 2 months. A Wilcoxon rank‐sum test was used to compare baseline biomarkers and changes in biomarkers from C1D1 to C1D8 between responders and nonresponders. A Bonferroni correction was used to adjust for multiple comparisons.

## 3 RESULTS

### 3.1 Patient characteristics

Six patients were enrolled during the dose escalation phase, three patients were enrolled at 120 mg daily, and an additional three were enrolled at 160 mg daily. There were no DLTs in either cohort. Therefore, the RP2D of regorafenib was identified as 160 mg daily. Ten additional patients were enrolled at this dose during the expansion phase.

Patient characteristics are described in Table 1. The median age was 74 (range 36–89), and 13 (81%) patients were male. Diagnoses included AML (*N* = 5, 31%, 3 de novo and two transformed from antecedent MDS), MDS (*N* = 7, 44%), CMML (*N* = 1, 6%), MF (*N* = 2, 13%), and MPN‐NOS (*N* = 1, 6%). Eleven (69%) patients had one prior line of therapy, four patients (25%) had two prior lines of therapy, and one patient (6%) had ≥3 prior lines of therapy. Within the MDS patients, three patients (42.9%) were high or very‐high risk by international prognostic scoring system (IPSS) criteria, and both MF patients were intermediate‐2 or high‐risk by dynamic international prognostic scoring system (DIPSS) criteria.

**TABLE 1:** Patient baseline characteristics. Baseline characteristics for patients enrolled in phase 1 study of regorafenib in advanced myeloid malignancies

Patients	*N* = 16 (%)
Age (median, range)	74 (36–89)
Sex (% male)	13 (81.3%)
Diagnosis	
De Novo AML	3 (18.8%)
Secondary AML^a^	2 (12.5%)
MDS	7 (43.8%)
MPN^b^	3 (18.8%)
CMML	1 (6.25%)
Risk stratification	
MDS	
High or very high	3 (42.9%)
Intermediate	2 (28.6%)
Low	2 (28.6%)
MF	
Int‐2 or High	2 (12.5%)
Int‐1 or Low	0
Prior lines of therapy	
1	11 (68.8%)
2	4 (25%)
≥3	1 (6.3%)
RAS mutation	5 (31.3%)

Abbreviations: AML, acute myeloid leukemia; CMML, chronic myelomonocytic leukemia;

MDS, myelodysplastic syndrome; MF, myelofibrosis; MPN, myeloproliferative neoplasms.

a Secondary AML arising from antecedent MDS.

b 1 MPN NOS and 2 MF.

All patients have discontinued study therapy, including five (31%) due to progressive disease and two (12.5%) due to unacceptable toxicity. Dose modifications and delays occurred in four and two patients, respectively. The median duration of treatment was 52.5 days or 1.85 cycles (interquartile range (IQR): 19–100 days)

All patients had next generation sequencing from bone marrow performed on 92 commonly mutated loci across 23 genes associated with hematologic malignancies, as described previously [23]. Five patients had pathogenic mutations in the *RAS* family, including two secondary AML patients with an *HRAS* and *KRAS* mutation, one AML patient with a *KRAS* mutation, one MDS patient with a *KRAS* mutation, and one MPN‐NOS patient with an *NRAS* mutation.

### 3.2 Safety

At the 120‐mg dose level, a single grade 3 event occurred and was an episode of preseptal cellulitis treated with intravenous antibiotics, possibly attributed to drug. There were no hematologic toxicities or liver function test liver function tests (LFT) abnormalities noted at the 120‐mg dose level. In the 160‐mg dose level and expansion cohort, approximately 20% of events were grade 3 or higher. The most common grade 3 or higher adverse effects were LFT abnormalities, fatigue, anemia, thrombocytopenia, and neutropenia, including two incidents of febrile neutropenia. Among the 16 patients enrolled, nine reported fatigue including three grade 3 AEs; eight patients had LFT abnormalities including three grade 3 AEs; and five patients reported anorexia (one grade 3 AE). Hematologic toxicities included anemia (three of 16 patients, of which one was grade 3), neutropenia (two of 16 patients, all were grade 3), and thrombocytopenia (four of 16 patients, of which three were Grade 3–4). Two patients discontinued regorafenib therapy due to unacceptable toxicity. No grade 3 or higher events occurred in cycle 1. One of these patients had a diagnosis of MPN‐NOS with an *NRAS G12D* mutation and experienced an increase in white blood cell (WBC) from 54 × 10^9^/L to 92 × 10^9^/L with 3.5% blasts within a week of starting regorafenib, which resolved after stopping the treatment. A follow‐up bone marrow biopsy showed no disease progression. Adverse effects are summarized in Table 2.

**TABLE 2:** Adverse events. Adverse events for patients enrolled in phase 1 study of regorafenib in advanced myeloid malignancies

	Dose level 1		Dose level 2		Escalation	
	Any grade	Any grade ≥ 3	Any grade	Any grade ≥ 3	Any grade	Any grade ≥ 3
Any event	16 (94.1%)	1 (5.9%)	40 (76.9%)	12 (23.1%)	59 (78.7%)	16 (21.3%)
Nonhematologic Toxicity						
LFT abnormalities	0	0	15 (37.5%)	5 (41.7%)	14 (23.7%)	1 (6.2%)
Fatigue	2 (12.5%)	0	3 (7.5%)	1 (8.3%)	12 (20.3%)	4 (25.0%)
Diarrhea	0	0	1 (2.5%)	0	0	0
Nausea	1 (6.3%)	0	0	0	2 (3.4%)	0
Constipation	0	0	0	0	2 (3.4%)	0
Anorexia	1 (6.3%)	0	1 (2.5%)	0	6 (10.2%)	2 (12.5%)
Infection	1 (6.3%)	1 (100%)	0	0	0	0
Fever	1 (6.3%)	0	1 (2.5%)	0	0	0
Rash	0	0	0	0	1 (1.7%)	1 (6.2%)
Mucositis	0	0	2 (5.0%)	0	0	0
Arrythmia	0	0	0	0	0	0
Muscle weakness	0	0	1 (2.5%)	0	4 (6.8%)	3 (18.8%)
Hoarseness	1 (6.3%)	0	2 (5.0%)	0	3 (5.1%)	0
Other	9 (56.3%)	0	7 (17.5%)	1 (8.3%)	9 (15.3%)	1 (6.2%)
Hematologic toxicity						
Neutropenia	0	0	3 (7.5%)	3 (25.0%)	1 (1.7%)	1 (6.2%)
Anemia	0	0	1 (2.5%)	0	2 (3.4%)	1 (6.2%)
Thrombocytopenia	0	0	3 (7.5%)	2 (16.7%)	3 (5.1%)	2 (12.5%)

Abbreviation: LFT, liver function test.

### 3.3 Efficacy

**FIGURE 1:**
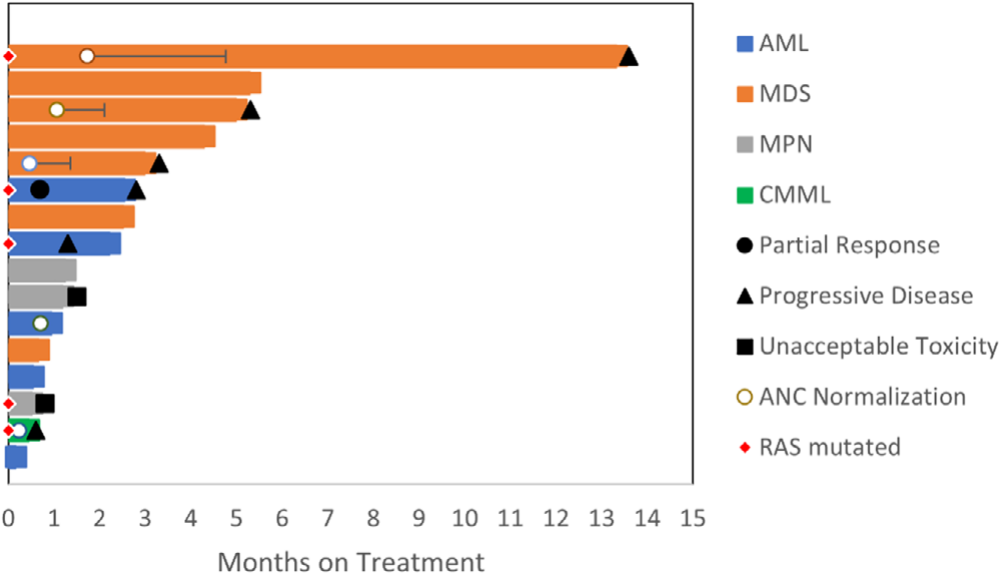
Patient responses. Swimmer's plot of responses to regorafenib in patients enrolled in phase 1 study of regorafenib in advanced myeloid malignancies

Figure 1 displays a swimmer's plot of patient responses to regorafenib. Of the 16 patients, best overall disease response included partial response in one AML patient and stable disease in 13 patients. Progressive disease was seen in two patients while on therapy. Median duration of treatment was 52.5 days (range: 8–437). One MDS patient had stable disease for 14 months on regorafenib before disease progression. The median overall survival for all patients on trial was 6.1 months (95% confidence interval (CI): 3‐NA).

We observed a modest improvement in cell counts in five patients with MDS and cytopenia, although this improvement did not meet IWG criteria for hematologic improvement given the short durations. Normalization of absolute neutrophil count occurred in all five patients, which was generally short‐lived. No improvements in hemoglobin or platelet parameters were seen.

### 3.4 Biomarkers

**FIGURE 2:**
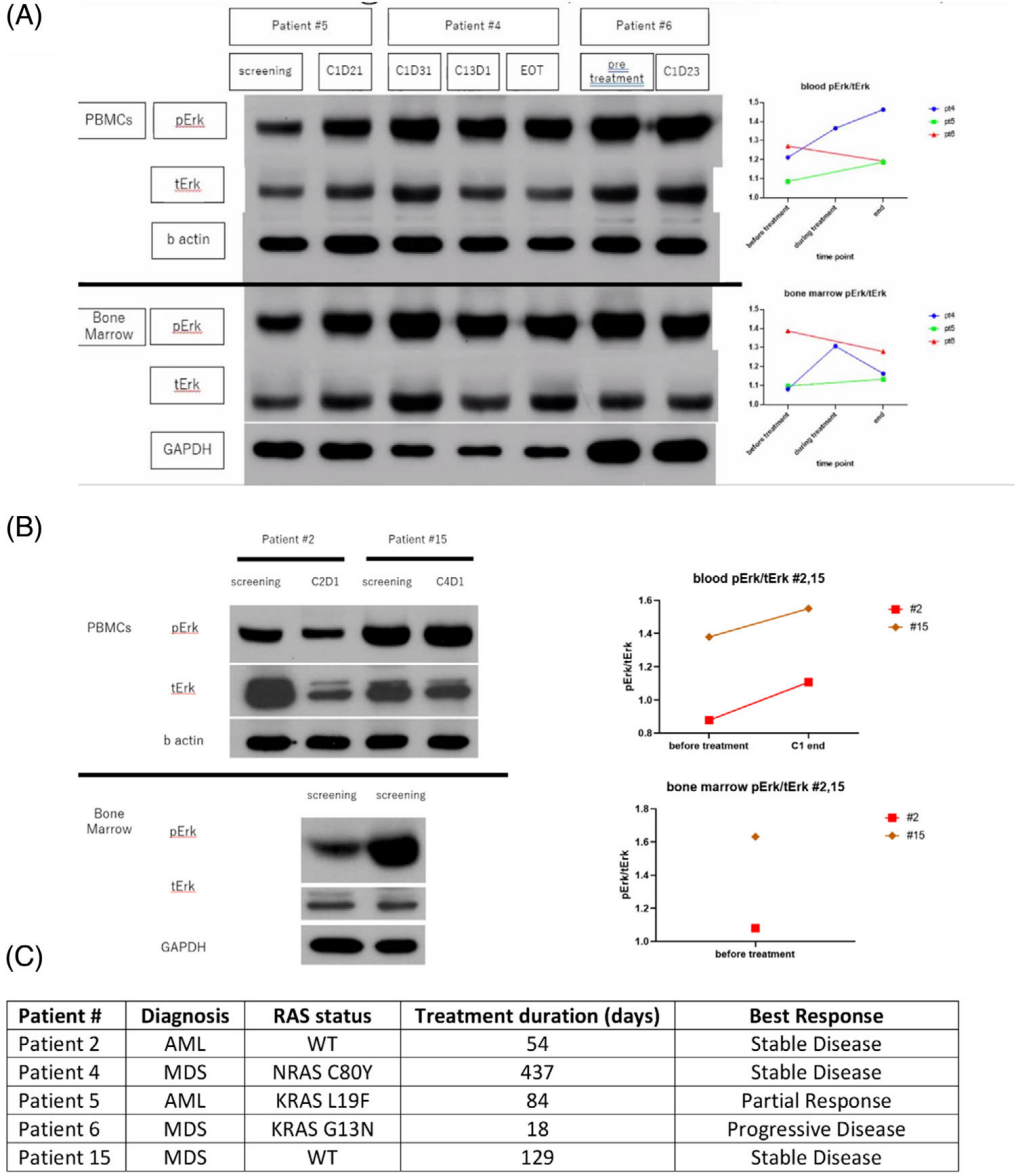
Western blotting demonstrating extra‐cellular signal regulated kinase (ERK) phosphorylation in three (A) RAS mutated and (B) RAS wild‐type patients during treatment. (C) Table listing patient characteristics

We first examined the changes in ERK phosphorylation (a downstream RAF factor) using bone marrow aspirates and PBMCs collected before and during regorafenib treatment from patients with RAS‐mutant disease (*n* = 3) and RAS‐wild type disease (*n* = 2). We found high levels of ERK activation in all samples, which were maintained after regorafenib treatment irrespective of RAS‐mutation status (Figure 2).

**FIGURE 3:**
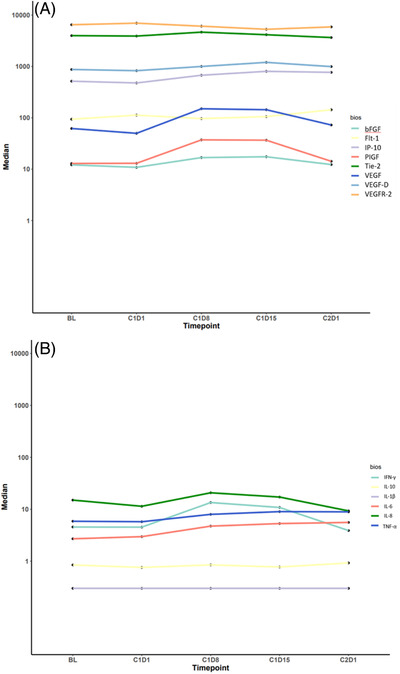
Changes in biomarkers. Spaghetti plot demonstrating changes in median (A) angiogenesis and (B) pro‐inflammatory biomarkers in advanced myeloid leukemia patients across time points during regorafenib treatment

We also examined the changes in soluble blood biomarkers (Figure 3). From C1D1 to C1D8, there were significant increases in median angiogenesis biomarker levels including VEGF (49.9–150.2, *p* = 0.04) and PIGF (13–37.2, *p* = 0.04), and a significant decrease in sVEGFR2 (6966.7 vs. 6085.8, *p* = 0.03). Increases in plasma sTIE‐2 and VEGF‐D were not significant when corrected for multiple comparisons. Pro‐inflammatory biomarkers that showed significantly increased concentration from C1D1 to C1D8 included TNFα (5.8–8, *p* = 0.03). There were also increases in plasma IL‐8, IL‐6, and IFN‐γ but these changes were not statistically significant when adjusted for multiple comparisons.

There were no significant associations between biomarkers at C1D1 and clinical response. An increase in plasma IL‐8 from C1D1 to C1D8 was associated with improved regorafenib response (*p* = 0.03), although this did not retain significance when corrected for multiple comparisons.

Finally, we examined the changes in cellular blood biomarkers after regorafenib treatment (Table 3). We found a decrease in the fraction of circulating CD3^+^/CD4^+^/CD8^–^ helper T‐cells from C1D1 to C1D8 (59.3–50.6, *p* = 0.02), although this was not significant when corrected for multiple comparisons. We detected no other significant changes in the fractions of circulating lymphocyte populations.

**TABLE 3:** Circulating lymphocyte populations. Median and interquartile ranges of circulating lymphocyte populations fractions by flow cytometry during regorafenib treatment in for patients with advanced myeloid malignancies. Units are in percentage of CD3^+^ total lymphocytes, and total CD3^+^ percentage is out of gating of all peripheral blood mononuclear cells

	C1D1	C1D8	C1D15	C2D1		
Cellular biomarkers	Median (IQR)	Median (IQR)	Median (IQR)	Median (IQR)	*p*‐value*	*p*‐value†
Total CD3^+^ (lymphocytes)	38.6 (21.4–51.2)	25.2 (16.8–47.3)	22.8 (9.1–40.4)	25.5 (22.5–29.1)	0.72	0.1
CD3^+^CD4^+^CD8^–^ (helper T‐cells)	59.3 (45.2–67.6)	50.6 (41.8–60.2)	55.5 (46.2–62.7)	61.6 (47.5–64.5)	0.02	0.72
CD3^+^CD4^+^CD8^–^CD25^+^ (regulatory T‐cells subset)	2.9 (0.9–5.3)	1.3 (0.3–4.4)	2.5 (0.7–6.2)	1.8 (0.2–4.4)	0.12	0.91
CD3^+^CD4^+^CD8^–^CD25^+^CD127^–^ (regulatory T‐cells subset)	1.1 (0.5–2.3)	0.6 (0.2–1.9)	0.9 (0.6–2.0)	0.6 (0.2–1.5)	0.11	0.53
CD3^+^CD4^+^CD8^–^CD25^–^CD127^+^ (Naïve or memory T cells)	23.4 (0.0–44.8)	16.4 (0.0–39.7)	26.2 (0.0–37.5)	33.6 (0.0–45.4)	0.79	0.58
CD3^+^CD4^+^CD8^–^CD25^–^CD127^–^ (regulatory T cells subset)	17.1 (11.0–44.6)	19.7 (10.9–41.7)	14.8 (11.9–43.8)	23.2 (15.7–39.8)	0.04	0.94
CD3^+^CD8^+^CD4^–^(cytotoxic T‐cells)	24.4 (16.2–34.2)	23.7 (14.5–39.8)	22.9 (10.8–34.9)	18.3 (9.5–25.4)	0.64	0.34
CD3^+^CD56^+^ (natural killer (NK) T‐cells)	0.7 (0.3–2.2)	0.4 (0.1–0.8)	0.2 (0.1–1.0)	0.2 (0.1–0.8)	0.53	0.23
CD3^–^CD56^+^ (NK cells)	3.8 (1.1–7.2)	2.6 (0.4–8.2)	1.6 (0.7–3.5)	1.7 (0.7–5.0)	1	0.73
CD3^+^CD8^+^CD4^–^CD25^+^ (regulatory T‐cells subset)	0.1 (0–0.2)	0 (0–0.1)	0.1 (0–0.2)	0.1 (0–0.2)	0.08	0.55

* C1D1 versus C1D8.

† C1D1 versus C1D15.

## 4 DISCUSSION

We report the first clinical trial to evaluate feasibility and biomarkers of regorafenib in patients with advanced myeloid malignancies. Regorafenib is an oral multikinase inhibitor that is food and drug administration (FDA) approved for treatment of multiple solid tumor types and has broad inhibitory activity of multiple kinases involved in proliferation, tumor immunity, and the tumor microenvironment. This makes regorafenib an appealing drug candidate in advanced myeloid malignancies, which is characterized by abnormalities in multiple signaling pathways.

We found that regorafenib was well tolerated at a starting dose of 160 mg daily. Phase I data showed no DLTs and mostly grade 1/2 AEs. LFT abnormalities, fatigue and anorexia occurred frequently in our study, consistent with reported adverse events with regorafenib in other tumor types [24, 25, 26]. Hematologic toxicity was also similar compared to other safety data [24, 25, 26]. However, hematologic events tended to be more severe, and attribution was difficult because of the underlying diseases being treated.

Interestingly, one *NRAS*‐mutated MPN patient experienced hyperleukocytosis with an accompanying increase in peripheral blasts within 1 week of starting regorafenib. This normalized after discontinuing therapy, and follow‐up bone marrow biopsy showed no evidence of disease progression. In addition to anti‐angiogenic effects, regorafenib has broad inhibitory activity in the RAS/RAF/MEK/ERK (MAPK) signaling pathway. Paradoxical activation of the MAPK pathway with RAF inhibition, as evidenced by ERK activation, has been documented in the presence of oncogenic RAS mutations, including worsening of an occult *Ras‐*mutated CMML in a melanoma patient treated with vemurafenib [27, 28]. However, none of the patients in this cohort, including five with *RAS* mutations, experienced signs of leukemic disease proliferation, and one patient with *RAS‐*mutated MDS remained on regorafenib for over a year with stable disease. We also did not see any signals for an association between *RAS* mutation status and disease response or AEs. However, further evaluation of regorafenib in *RAS‐*mutated leukemias should include close hematologic monitoring for signs of disease acceleration caused by potential paradoxical activation of ERK signaling upon RAF inhibition. Interestingly, we found no inhibition of MAPK pathway in samples from three *RAS* mutant or two *RAS* wild‐type cases. Moreover, of these three patients, one had prolonged stable disease on regorafenib for over 1 year, and one patient had initial partial response, suggesting that MAPK activation may not be the mechanism of benefit or indicate regorafenib treatment failure.

The results of the pharmacodynamic biomarker studies were consistent with regorafenib's known anti‐angiogenic activity. We did not find associations between baseline angiogenic or proinflammatory cytokines with clinical outcomes when correcting for multiple comparisons. However, we found an increase in IL‐8 was associated with clinical response. IL‐8 is an inflammatory chemokine that has known associations with disease severity and overall survival in advanced myeloid malignancies such as MF [29]. Its significance in the context of regorafenib therapy in AML is unclear and should be further investigated. Our flow‐cytometry‐based analyses also demonstrated decreases in helper T cell populations with regorafenib therapy and no other changes seen in other examined lymphocyte populations. In this small and heterogeneous cohort, our correlative analysis was exploratory and hypothesis generating.

Although the primary objective of our study was safety, we found evidence for limited clinical activity of regorafenib in this advanced population as most patients experienced stable disease. The median duration of treatment was relatively short at approximately two cycles, with some lower‐risk MDS patients showing more durable responses, including one for over 1 year. These responses are consistent with regorafenib's overall benefits of disease stabilization. Studies of regorafenib in hepatocellular carcinoma for instance have noted overall survival (OS) improvement with few complete responses, including reduction of arterial enhancement and increased central tumor necrosis without changes in tumor volume [24, 30]. Greater anti‐leukemic disease activity may be achieved with using regorafenib as a component in combination therapy, such as with immune checkpoint blockade. While immune checkpoint inhibitors have shown some clinical activity in myeloid malignancies, overall results have been less impressive especially in comparison to solid tumors with high immunogenicity and mutation rates. Immunologic priming may be required for checkpoint inhibitors to be effective, and combination therapies of regorafenib and anti‐PD1 therapy have shown promise in solid tumor settings [31, 32].

In conclusion, phase I data showed that regorafenib has an acceptable safety profile in relapsed/refractory myeloid malignancy patients, a population where few treatment options exist. Regorafenib induced stable disease in most patients and modest improvements in cell counts in MDS patients. Future studies should investigate which leukemic subpopulations may derive benefit from regorafenib and evaluate the potential role of regorafenib in combination therapy.

## CONFLICT OF INTEREST

Amir T. Fathi: Consulting: Foghorn, Amgen, Celgene, BMS, Agios, Novartis, Ipsen, Forma, Abbvie, Genentech, Morphosys, and Takeda. Donna Neuberg: Stock ownership in Madrigal Pharmaceuticals, and received research funding from Pharmacyclics. Yi‐Bin Chen: Consulting: Daiichi, Abbvie, Equilium, Incyte, Jasper, Celularity, and Actinium; clinical trial support: Abbvie, Celgene, BMS, Agios, and Servie; Dan G. Duda received consultant fees from Simcere, Surface Oncology, Sophia Bioscience, Innocoll and BMS, and research grants from Exelixis, BMS, and Surface Oncology. Dan G. Duda's research is supported by National Institutes of Health (NIH) (grant numbers: R01CA260857, R01CA247441, R01CA254351, R03CA256764, and P01CA261669) and Department of Defense PRCRP (grant numbers: #W81XWH‐19‐1‐0284 and W81XWH‐21‐1‐0738). Gabriela Hobbs: Consulting fees from Abbvie, Incyte, Blueprint, Novartis, and Keros; research support from Bayer, Incyte and Constellation; grants: ECOR PSDA Award and ASH‐AMFDP award. The other authors declare no potential conflict of interest. Result of this study has previously been reported as an abstract at the 2020 American Society of Hematology Annual Meeting (Virtual). The funders had no role in data analysis or the preparation of this article.

## AUTHOR CONTRIBUTIONS

Joan How wrote the manuscript. Siyang Ren analyzed and interpreted data and performed statistical analysis. Jennifer Lombardi‐Story, Meghan Bergeron, Julia Foster, Phillip C. Amrein, Andrew M. Brunner, Amir T. Fathi, Hanno Hock, Jenna Moran, Rupa Narayan, Aura Ramos, Tina Som, and Meghan Vartanian performed research and collected data. Joan How, Anna Khachatryan, Hiroto Kikuchi, and Mei Rosa Ng performed research, collected, analyzed, and interpreted data. Yi‐Bin Chen, Dan G. Duda, and Gabriela Hobbs designed and performed research, collected, analyzed, and interpreted data.

## ETHICS STATEMENT

All procedures followed were in accordance with the ethical standards of the responsible committee on human experimentation (institutional and national) and with the Helsinki Declaration of 1975.
